# Supplementary material for: A human model to deconvolve genotype-phenotype causations in lung squamous cell carcinoma
Source: Nat Commun. 2025 Apr 4;16:3215. doi: 10.1038/s41467-025-58343-y (PMC11971459; doi:10.1038/s41467-025-58343-y)
Supplement: Supplementary file 1 — Supplementary Information [file 41467_2025_58343_MOESM1_ESM.pdf]

SUPPLEMENTARY INFORMATION

A human model to deconvolve genotype-phenotype causations in lung squamous cell carcinoma

Julia Ogden[1], Robert Sellers[1], Sudhakar Sahoo[1], Anthony Oojageer[1], Anshuman Chaturvedi[2], Caroline Dive[1, 3, 4], Carlos Lopez-Garcia[1, 4, 5]

[1]Cancer Research UK Manchester Institute, Wilmslow Road, M20 4BX, Manchester (United Kingdom)

[2]Department of Histopathology, The Christie Hospital, Wilmslow Road, Manchester, M20 4BX (United Kingdom)

[3]Cancer Research UK, National Biomarker Centre, Wilmslow Road, M20 4BX, Manchester (United Kingdom)

[4]Cancer Research UK Lung Cancer Centre of Excellence, Wilmslow Road, M20 4BX, Manchester (United Kingdom)

[5]Corresponding author: carlos.lopezgarcia@cruk.manchester.ac.uk

Contents: Supplementary Figures 1-9, Supplementary Tables 1-2 and the legends for Supplementary Data files 1-7.

SUPPLEMENTARY FIGURES

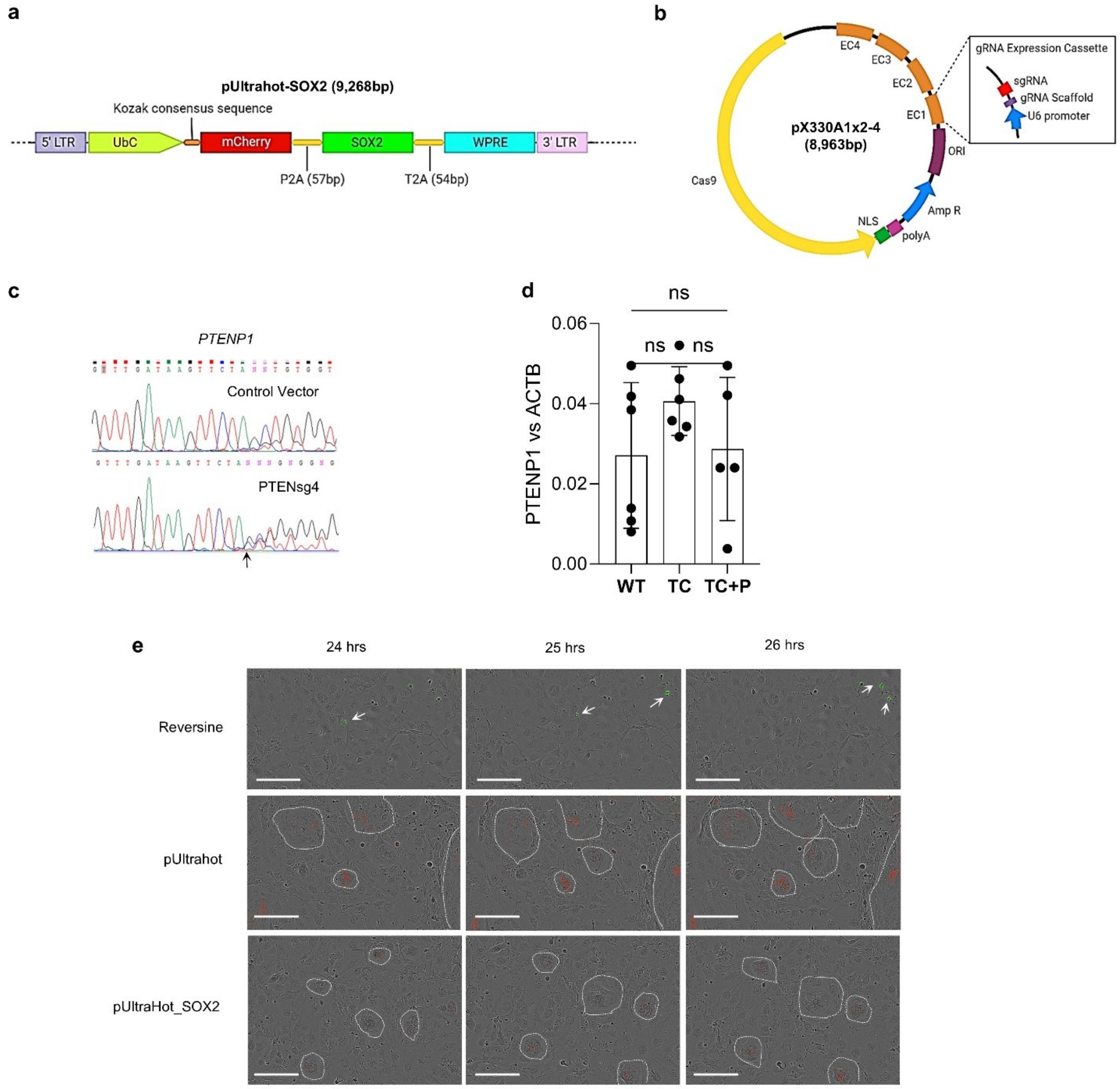

Supplementary Figure 1: Parental vectors used in this work, *PTEN* CRISPR off-target effect, and effect of SOX2 on hBEC viability.
a. Diagram of pUltrahot construct insert region with mCherry reporter and SOX2 cDNA intersected by a P2A self-cleaving peptide sequence. **b**. Diagram of the pX330 multiplex CRISPR/Cas9 vector used to generate tumour suppressor knockouts. The vectors contained a maximum of four gRNA sequences inserted into individual gRNA expression cassettes (EC). **c**. Sanger sequencing traces of the *PTENP1* locus from hBECs electroporated with the empty pX330 CRISPR/cas9 vector (control vector) and those electroporated with a px330 CRISPR/cas9 vector containing the PTEN gRNA #4. **d.** qPCR analysis of *PTENP1* RNA expression in wildtype, TC and TC+P mutants. Data is shown as the mean of 6 or 5 (for the TC+P mutant) independent ALI cultures +/-SD. Adj.P values were calculated by one-way ANOVA with multiple comparisons and Tukey's *post hoc* test **e**. Representative images of cleaved caspase 3 apoptosis assays depicting live cell imaging of hBEC cultures transduced with the empty pUltrahot vector, or pUltrahot with SOX2 cDNA (pUltrahot_*SOX2*). Imaging was carried out every 60 minutes from timepoints 0 to 96-hours. Images show 24-, 25- and 26-hour timepoints. hBECs treated with reversine acted as an apoptosis positive control. hBEC colonies are identified by dashed white lines and mCherry expressing hBECs are shown with red fluorescence. Cells undergoing apoptosis appear in green and are highlighted by white arrows. Scale bars = 200µm. Source data are provided as a Source Data file.

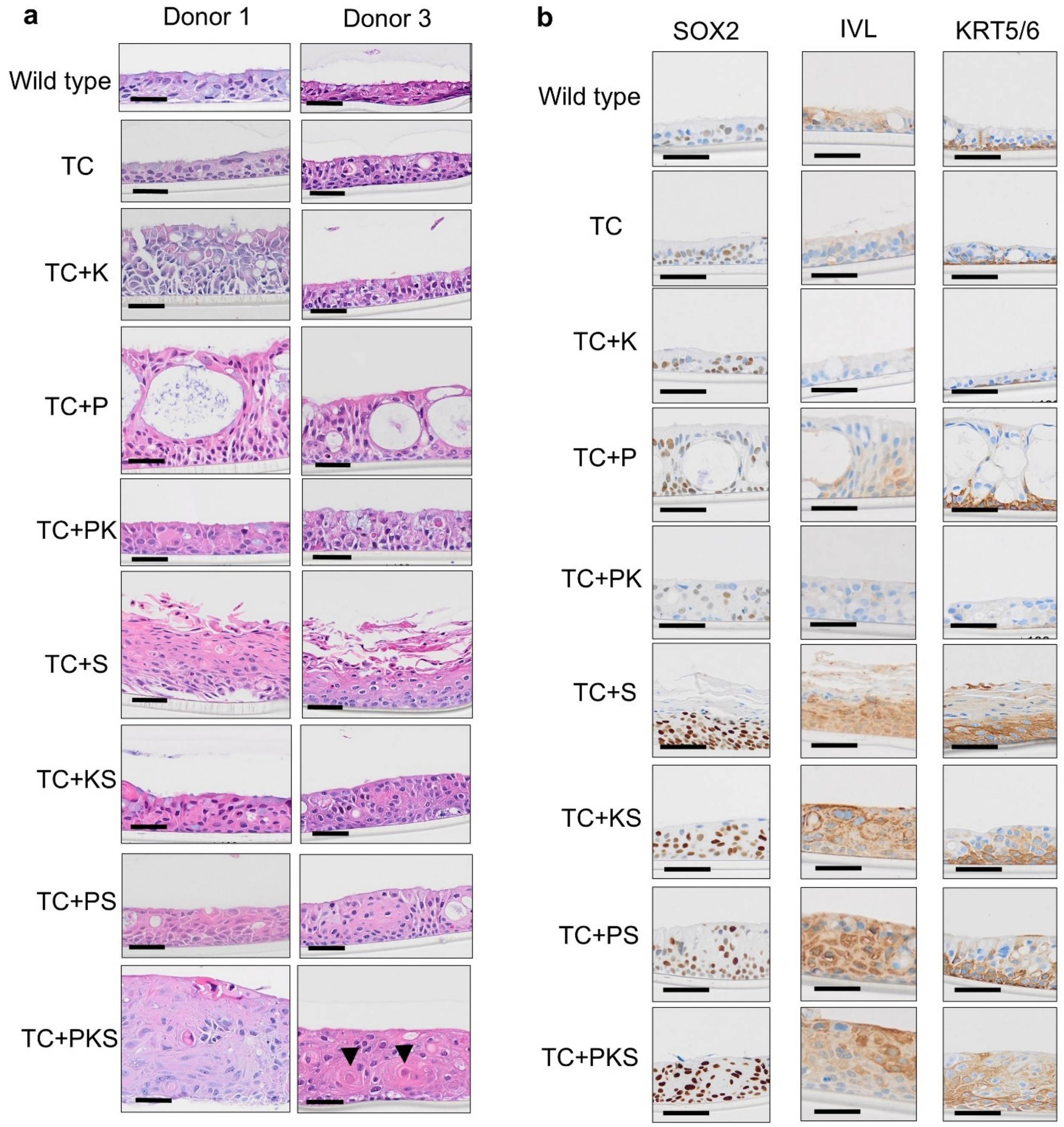

Supplementary Figure 2: Histological analysis of ALI cultures generated with mutant hBECs from donors 1 and 3.

a. Representative haematoxylin and eosin stained formalin fixed paraffin sections showing the histology of ALI cultures generated using mutant hBECs from donor 1 and 3. Arrowheads show areas of keratinisation. Scale bars = 50μm. **b**. Representative images of **i**mmunohistochemistry staining of ALI culture formalin fixed paraffin embedded sections to detect the expression of SOX2 and proteins associated with terminal squamous differentiation in donor 2. IVL = involucrin, KRT5/6 = cytokeratin 5/6. Scale bars = 50μm.

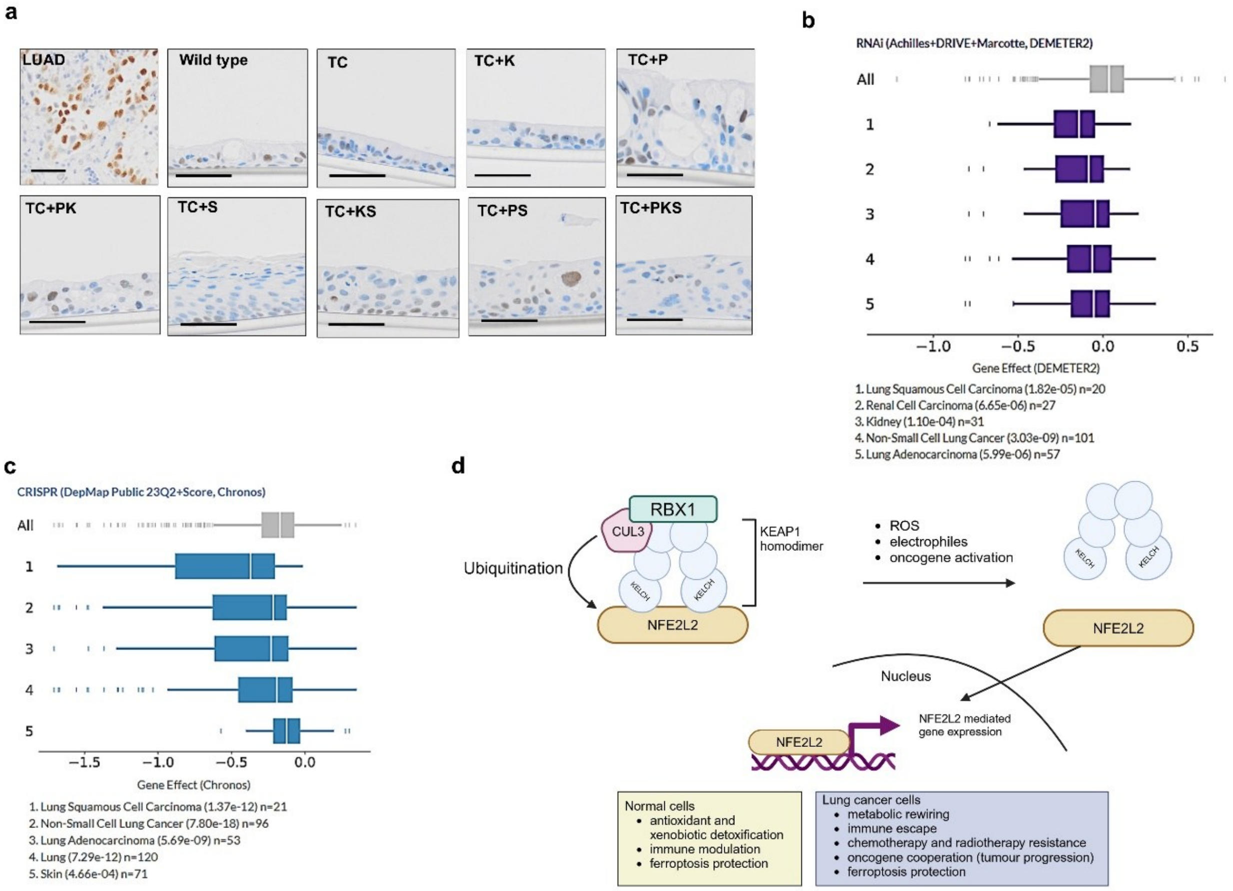

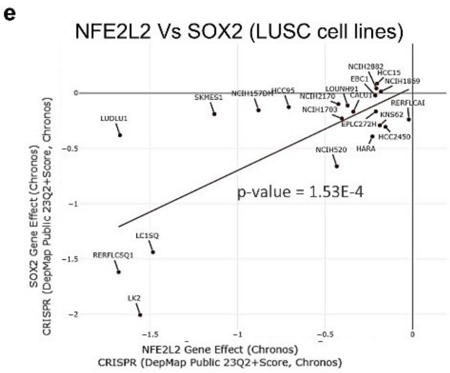

f

Top 10 Co-Dependencies

Gene	NFE2L2 Correlation
SLC33A1	0.337498032
TAPT1	0.304182589
ADPGK	0.258318251
SUCO	0.25283235
SOX2	0.223331256
UXS1	0.209901334
RNF217	0.208573856
TXN	-0.205970165
TSC1	-0.194306972

Supplementary Figure 3: Expression of lung adenocarcinoma biomarkers in ALI cultures, and DepMap analysis of LUSC dependency on *NFE2L2* (NRF2) and *SOX2*.

a. Immunohistochemistry staining of ALI cultures for the pulmonary developmental transcription factor TTF-1. TTF-1 is positive in LUAD control samples but expressed at low levels in ALI cultures. Scale bars = 50µm. **b-c.** DepMap data showing cell line lineages ranked *NFE2L2* gene effect in both the RNAi (**b**) and CRISPR (**c**) datasets. Top 5 cancer types are shown. Boxes show median gene effect +/- upper and lower quartiles. Whiskers mark the 5% and 95% percentiles **d.** DepMap co-dependency data showing the top 10 significant genes correlated with *NFE2L2* expression. Genes are ranked by correlation coefficient. **e**. DepMap data showing the correlation between *NFE2L2* and *SOX2* gene effects in LUSC cell lines. *NFE2L2* and *SOX2* gene effects are significantly correlated (p-value = 1.53E-4). **f.** A schematic of the KEAP1-NRF2 pathway with examples of functional outputs in normal and cancer context. Panel 3d created in BioRender. Lopez-garcia, C. (2025) https://BioRender.com/j69e440.

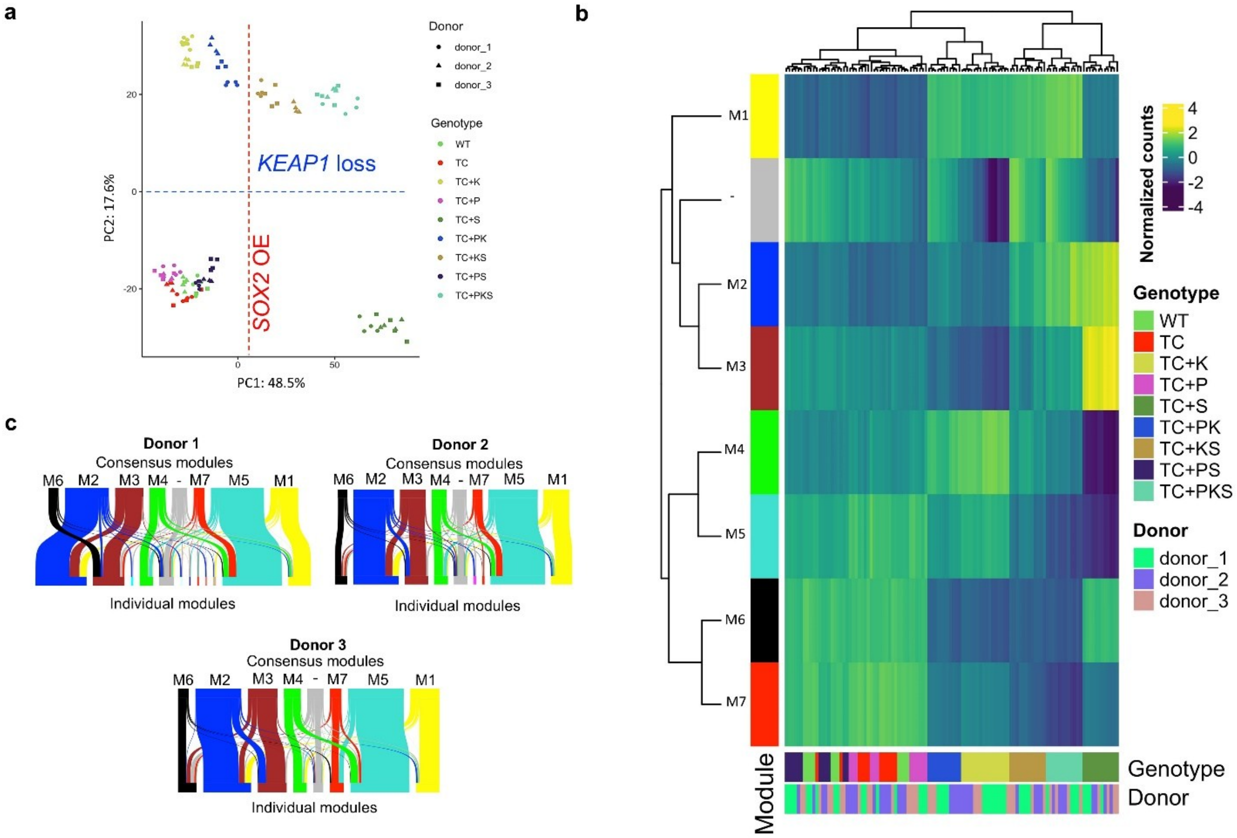

Supplementary Figure 4: Overall transcriptomic analyses of ALI cultures to estimate inter-donor and inter-genotype diversity.

a. Principal component analysis (PCA) using the top 500 variable genes from RNAseq of 108 ALI cultures. Four ALI cultures from each genotype were included, each from one of the of three hBEC donors. PC1 separated samples based on the presence or absence of *SOX2*^{OE} (red dotted line) and PC1 separated samples based on the presence or absence of functional *KEAP1* (blue dotted line). **b** Seven consensus modules of co-expressed genes were identified using weighted gene co-expression network analysis (WGCNA)**.** Modules are labelled M1-M7. Genes marked by the grey bar did not fit any specific co-expression module. Heatmap indicates the average normalised counts of module gene sets within each sample. Hierarchical clustering was performed based on both genotype and module expression. **c.** Sankey diagrams showing the relationship between the seven consensus modules (identified using all data) and the individual donor modules (identified separately for each donor) for donors 1-3.

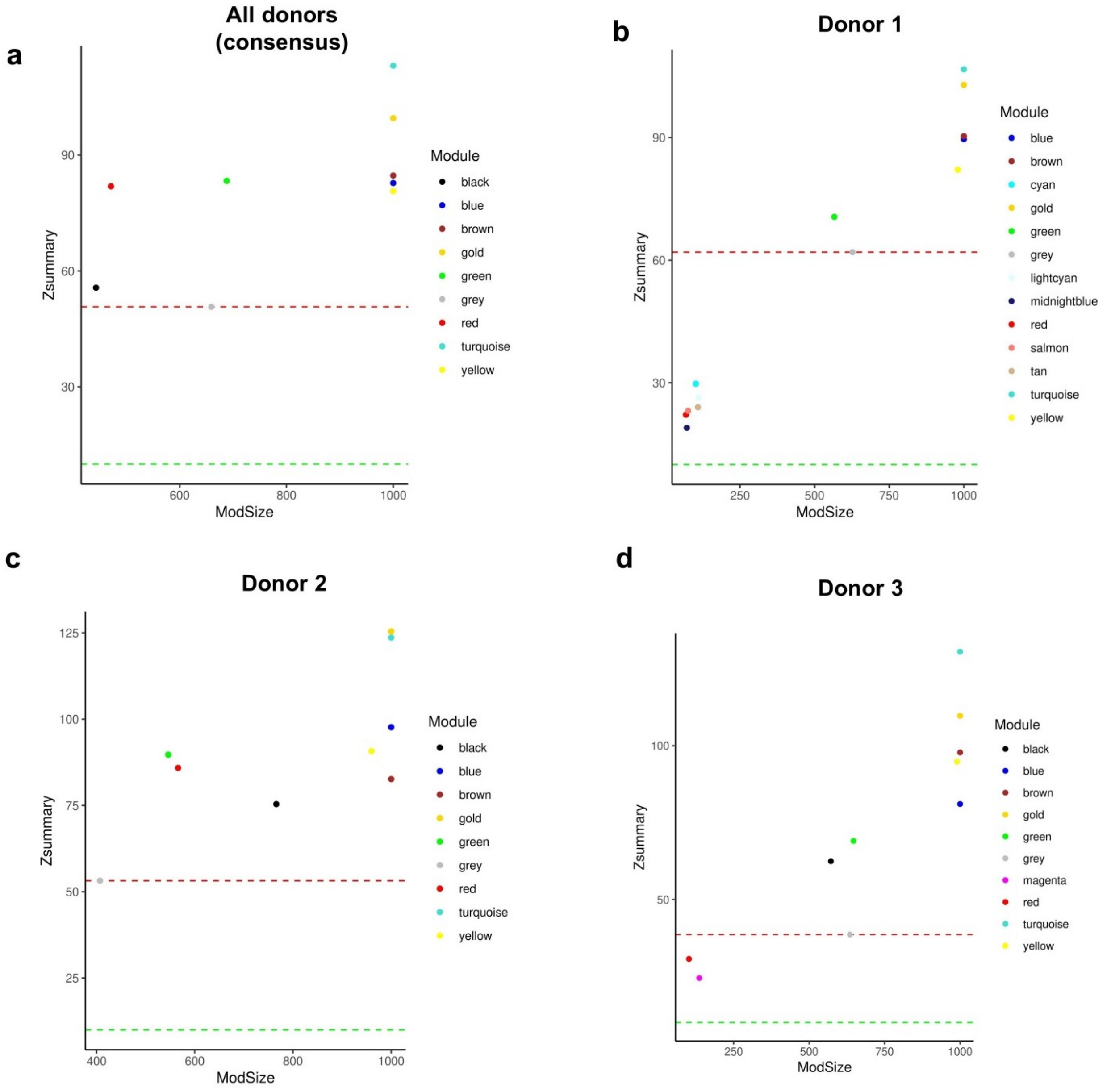

Supplementary Figure 5: Quality control analyses of the WGCNA modules.

a-d. Zsummary plots for WGCNA modules for consensus modules **(a)** and individual donor modules **(b-d)**. High Zsummary values represent high stability. The red dotted lines represent the Zsummary intercept of the grey module (genes which failed to be classified). The green dotted line represents Zsummary=10, he minimal recommended threshold for evidence of conservation.

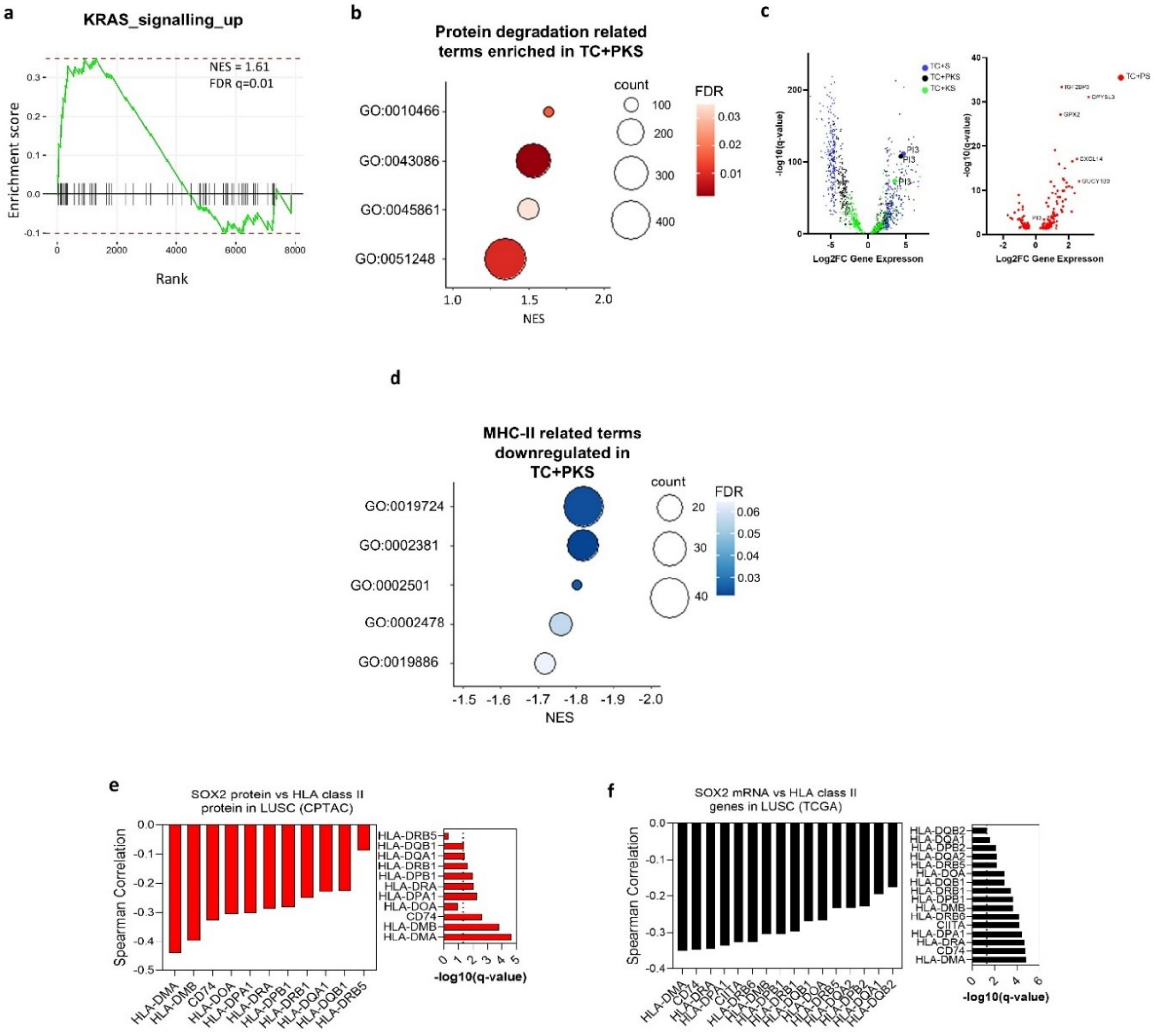

Supplementary Figure 6: Expanded analysis of SOX2-regulated biological processes.

a. Gene set enrichment analysis showing the significant positive enrichment (q-value<0.05) of the KRAS signalling up hallmark (MSigDB, H: hallmark gene sets) in genes differentially expressed in TC+PKS versus TC (q-value<0.05). **b.** Gene set enrichment analysis with dot plot showing significant (q-value<0.05) positively enriched gene ontology terms related to protein degradation within genes differentially expressed (q-value < 0.05, log2FC in the TC+PKS versus TC comparisons. GO:0010466=negative regulation of peptidase activity, GO:0043086=negative regulation of catalytic activity, GO:0045861=negative regulation of proteolysis, GO:0051248=negative regulation of protein metabolic process. **c.** Volcano plots of differentially expressed genes in all *SOX2*^{OE} mutants versus TC mutants showing *PI3* expression regulation. **d.** Gene set enrichment analysis with dot plot showing significant (q-value<0.05) negatively enriched gene ontology terms related to antigen processing and presentation within genes differentially expressed (q-value<0.05) in the TC+PKS versus TC comparisons. GO:0019886=antigen processing and presentation of exogenous peptide antigen via MHC class II, GO:0002501=peptide antigen assembly with MHC protein complex, GO:0002478=antigen processing and presentation of exogenous peptide antigen, GO:0002381=immunoglobulin production involved in immunoglobulin mediated immune response, GO:0019724=B cell mediated immunity. **e-f.** Spearman correlations of genes associated with MHC-II antigen progressing and presentation with SOX2. Analysis was performed in cBioportal using LUSC samples from the CPTAC (protein, 80 samples) and TCGA (mRNA, 178 samples) datasets. Left panels show -log10 (q-value) and dashed line indicates significance thresholds (q-value<0.05). p-values were calculated using 2-sided t-tests and q-values were calculated using the Benjamini-Hochberg FDR correction procedure. Source data are provided as a Source Data file.

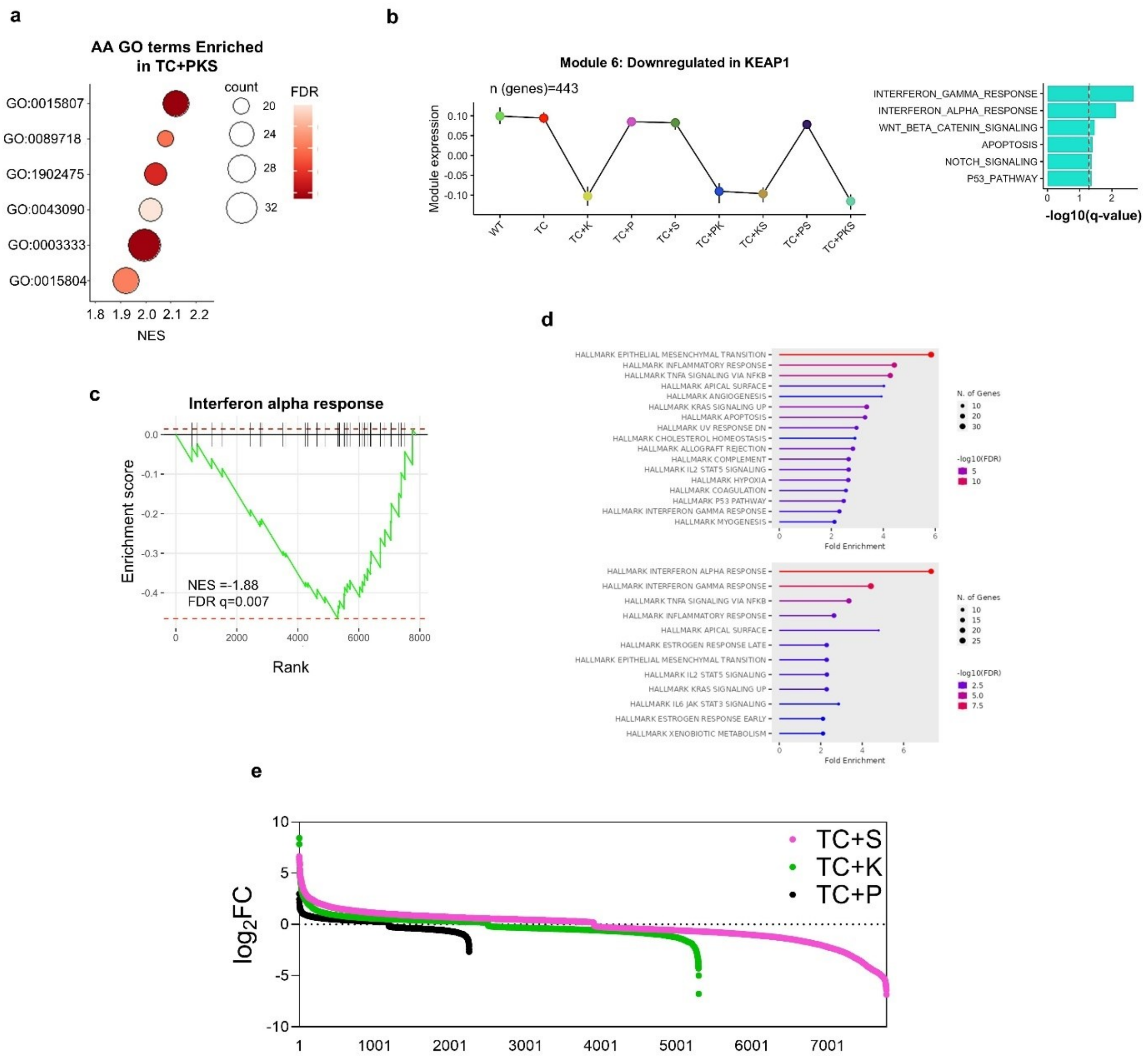

Supplementary Figure 7: Expanded analysis of OSR-regulated biological processes and distribution of gene expression fold-changes for each pathway.

a. Gene set enrichment analysis with dot plot showing significant (q-value<0.05) positively enriched gene ontology terms related to amino acid transport within genes differentially expressed (q-value< 0.05) in the TC+PKS versus TC comparisons. GO:0015807=amino acid transport, GO:0003333=amino acid transmembrane transport, GO:1902475=alpha amino acid transmembrane transport, GO:0089718= amino acid import across plasma membrane, GO:0015804=neutral amino acid transport, GO:0043090= amino acid import. **b**. Left panel shows line graph depicting the expression of consensus module 6 in all hBEC mutants, showing that the expression dips in all *KEAP1* disrupted mutants. Right panel shows overrepresentation analysis for hallmarks (MSigDB, H: hallmark gene sets), carried out using module 6 genes. Dotted line indicates significance threshold (q-value<0.05). **c.** Gene set enrichment analysis showing the negative enrichment of interferon alpha response (top) and interferon gamma response (bottom) hallmarks (MSigDB, H: hallmark gene sets) in genes differentially expressed in TC+PKS versus TC (q-value<0.05). **d.** Hallmarks enrichment (MSigDB, H: hallmark gene sets) in genes differentially expressed in LUSC with alterations targeting the OSR pathway (q>0.05). These include mutations or copy-number changes in *NFE2L2*, *KEAP1* and *CUL3*. Differentially expressed genes were downloaded from the cBioportal CPTAC (protein, top) and TCGA (mRNA, bottom) databases and enrichment analysis was carried out using the shinyGO application (FDR<0.05). **e.** Log2FC distributions of genes differentially expressed compared to TC mutants following activation of each of the three pathways (q-value<0.05).

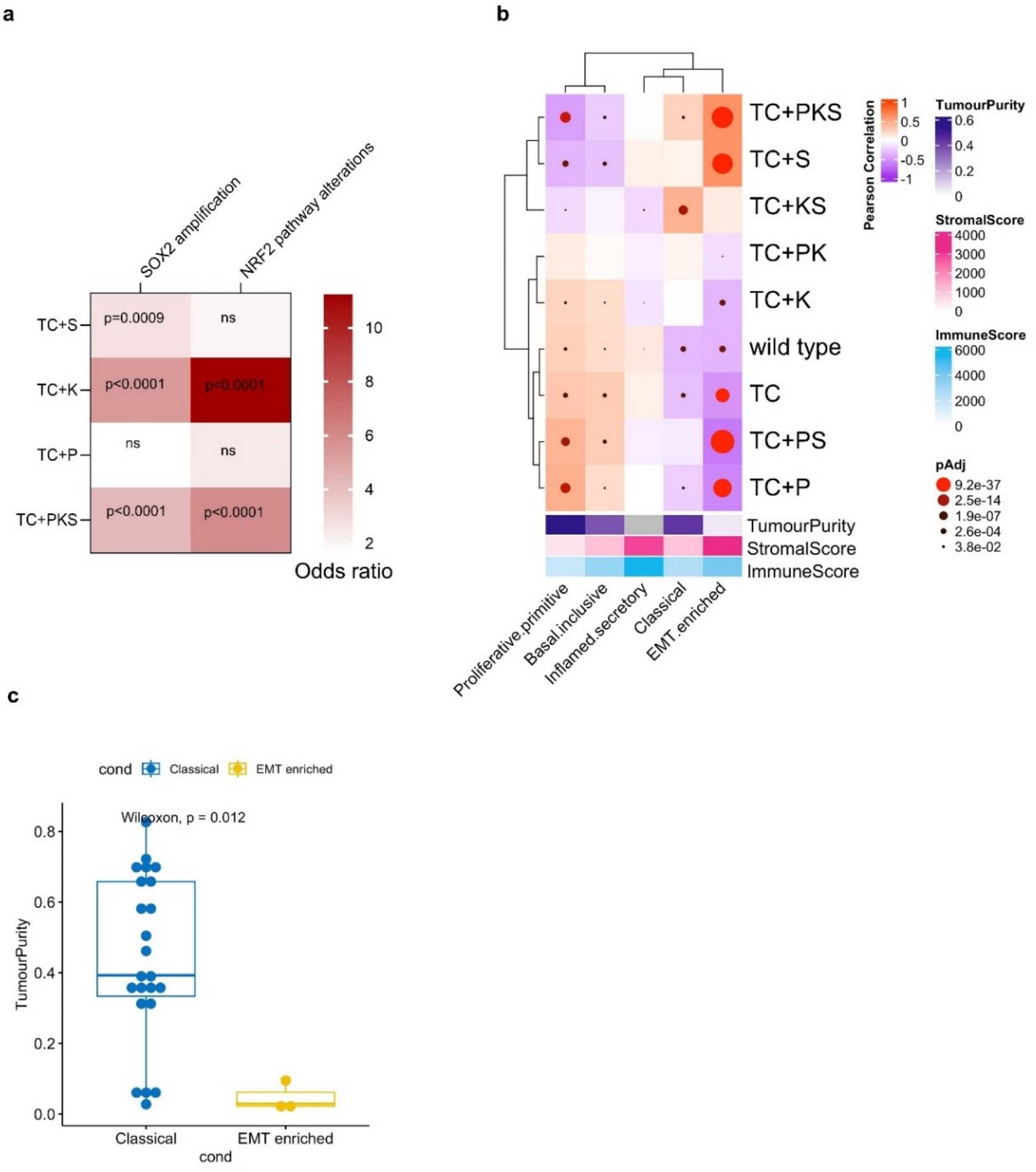

Supplementary Figure 8: Comparisons between gene-expression profiles in ALI cultures and LUSC samples.

a. Side-by-side comparison between GO Biological Process terms regulated individually by each pathway in ALI cultures and LUSC patient samples. The diagram shows the statistical significance of the overlap between enriched terms in the TC+S vs TC, TC+K vs TC, TC+P vs TC and TC+PKS vs TC comparisons and the terms significantly enriched in LUSC tumours (TCGA cohort, 178 samples) with and without *SOX2* amplification and with and without alterations in components of the OSR pathway (*NFE2L2*, *KEAP1* and *CUL3*). A similar analysis for *PTEN* mutations is not shown as the low number of *PTEN*-mutant LUSC samples resulted in only four significantly regulated GO Biological Process gene sets. The statistical significance of the overlap between the enriched GO terms in ALI cultures and LUSC samples was obtained by Chi-square test (df=1, CI=95%) against a background of GO BP gene sets represented by one or more differentially expressed in each relevant comparison. **b.** Pearson correlations of the enrichment scores (NES) of the GO Biological Processes significantly regulated by the SD, PI3K/Akt and OSR pathways simultaneously (TC+PKS vs TC comparison) in all mutants and the LUSC molecular subtypes described in the CPTAC cohort (80 samples). **c**. Tumour purity of the classical (n=24) and EMT (n=3) subtypes in the CPTAC cohort. Boxes show median tumour purity +/- upper and lower quartiles. Whiskers mark the 5% and 95% percentiles. *P* value was calculated by Wilcoxon test. Source data are provided as a Source Data file.

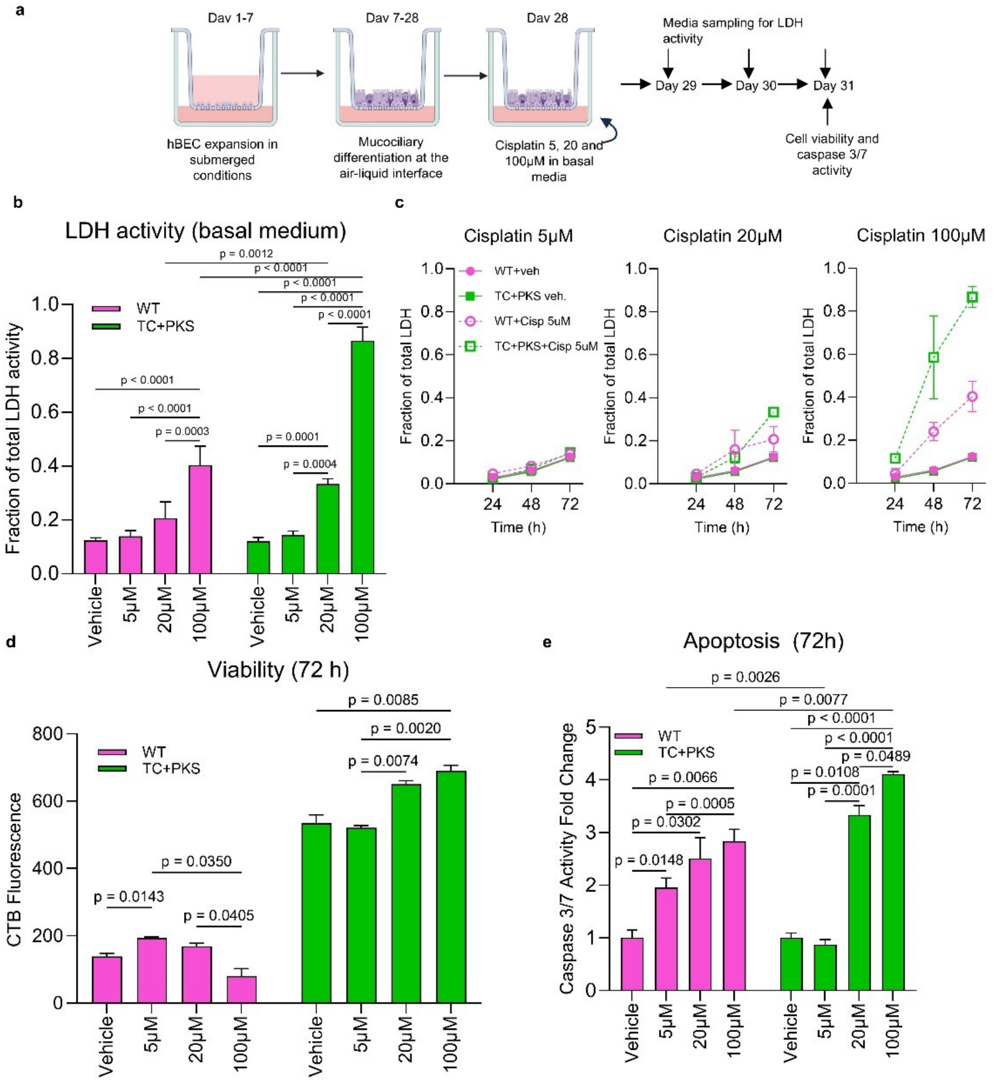

Supplementary Figure 9: Validation of read-outs to measure effect of chemotherapy in ALI cultures generated with wild type and TC+PKS mutants.

a. Experimental design to measure the effect of cisplatin treatment in wild type and TC+PKS ALI cultures. ALI cultures were seeded and differentiated using the normal protocol. 28 days after seeding, cisplatin was added to the basal media at 5, 20 and 100μM concentration and 10μl sample of media was taken 24, 48 and 72 hours after cisplatin addition to measure LDH activity. Caspase 3/7 activity and resazurin assays were carried out at 72 hours. **b.** LDH activity in basal media after 72 hours treatment with cisplatin measure with LDH-Glo™. LDH activity in the media was normalised to total cellular LDH activity in untreated ALI cultures samples. Total LDH activity was calculated in a cell lysate of untreated ALI cultures. Data is shown as the mean of 3 independent experiments +/-SEM. Adj.P values were calculated by two-way ANOVA with multiple comparisons and Tukey's *post hoc* test. **c**. Time course experiment showing the accumulated LDH activity in basal media after 24, 48, and 72 hours treatment with 5, 20 and 100μM cisplatin. **d.** Cell viability assay after 72h of cisplatin treatment in ALI cultures measured with CellTiter-Blue®. The reagent was added to the apical compartment of the ALI culture and incubated at 37°C for 2 hours. Data is shown as the mean of 3 independent experiments +/-SEM. Adj.P values were calculated by two-way ANOVA with multiple comparisons and Tukey's *post hoc* test. **e.** Fold change changes in caspase 3/7 activity after 72h of cisplatin treatment in ALI cultures measured with the Apo-One® assay. The Apo-One® reagent was added to the apical compartment of the assay and cell lysis was carried out for two hours before measuring fluorescence. Data is shown as the mean of 3 independent experiments +/-SEM. Adj.P values were calculated by two-way ANOVA with multiple comparisons and Tukey's *post hoc* test. Panel 9b created in BioRender. Lopez-garcia, C. (2025) https://BioRender.com/n05e704. Source data are provided as a Source Data file.

SUPPLEMENTARY TABLES

Donor number	Donor ID (Lonza)	Batch	Sex	Smoking status
Donor 1	36722	18TL290281	male	Non-smoker
Donor 2	28910	482214	male	Non-smoker
Donor 3	35211	18TL052522	female	Non-smoker

Supplementary Table 1: hBEC donor information provided by Lonza Biosciences. The age of the donors ranged from 62 to 75.

Name	Target	Exon	Sequence
sgRNA_*PTEN3*	*PTEN*	e5	AGAGGCCCTAGATTTCTATG
sgRNA_*PTEN4*	*PTEN*	e5	GGTTTGATAAGTTCTAGCTG
sgRNA_*PTEN5*	*PTEN*	e5	TGTGCATATTTATTACATCG
sgRNA_*PTEN6*	*PTEN*	e5	TTTGAAGACCATAACCCACC
sgRNA_*PTEN7*	*PTEN*	e5	ATGTGCATATTTATTACATC
sgRNA_*PTEN8*	*PTEN*	e7	AAGATATATTCCTCCAATTC
sgRNA_*PTEN9*	*PTEN*	e7	ATTCAGGACCCACACGACGG
sgRNA_*PTEN10*	*PTEN*	e7	ACACGACGGGAAGACAAGTT
sgRNA_*PTEN11*	*PTEN*	e7	CAATTCAGGACCCACACGAC
sgRNA_*PTEN12*	*PTEN*	e7	ACACGACGGGAAGACAAGTT
sgRNA_*CDKN2A3*	*CDKN2A*	e2	GCTGCTGCTGCTCCACGGCG
sgRNA_*CDKN2A4*	*CDKN2A*	e2	CGCCACTCTCACCCGACCCG
sgRNA_*CDKN2A5*	*CDKN2A*	e2	TCGCGATGTCGCACGGTACC
sgRNA_*CDKN2A6*	*CDKN2A*	e2	TGGGCCATCGCGATGTCGCA
sgRNA_*KEAP13*	*KEAP13*	e3	GGGCCGCCTGATCTACACCG
sgRNA_*KEAP14*	*KEAP14*	e3	AGGCTTACAACCCCAGTGA
sgRNA_*KEAP15*	*KEAP15*	e3	GCGTGCCCCGTAACCGCATCG
sgRNA_*KEAP16*	*KEAP16*	e2	CAGCCAGATCCCAGGCCTAG
sgRNA_*TP53*	*TP53*	e4	GGATGATTTGATGCTGTCCC

Supplementary Table 2: List of guide RNAs tested for the generation of CRISPR/Cas9 knock out cells. Guides used for final model generation are highlighted in yellow.
