# Supplementary material for: A human model to deconvolve genotype-phenotype causations in lung squamous cell carcinoma
Source: Nat Commun. 2025 Apr 4;16:3215. doi: 10.1038/s41467-025-58343-y (PMC11971459; doi:10.1038/s41467-025-58343-y)
Supplement: Supplementary file 3 — Description of Additional Supplementary Files [file 41467_2025_58343_MOESM3_ESM.pdf]

Description of Additional Supplementary Files

Supplementary Data 1: List of genes included in each WGCNA consensus module shown in Supplementary Figure 4. Each module is shown in separate tabs. P-values calculated from ANOVA comparison of full (~genotype + donor) and reduced (~donor) regression models, multi-test correction performed using Holm's method. Genes with pAdj ≤ 0.05 (CI 95%) were classed as s ignificantly affected by genotype and included within the WGCNA.

Supplementary Data 2: MSigDB Hallmark enrichments for each WGCNA consensus module shown in Supplementary Figure 4. Each module is shown in separate tabs. Statistically significant e nrichments (q<0.05) are highlighted in green. Overrepresentation analysis performed in R using enricher function from clusterProfiler.

Supplementary Data 3: Differentially expressed genes in TC+PKS, TC+S, TC+K and TC+P mutants relative to TC. Each tab contains one comparison. Differential expression (DE) test performed using DESeq2, only significantly DE genes (padj ≤ 0.05; CI 95%) included in table; raw a nd DESeq2-normalised counts attached.

Supplementary Data 4: GSEA analysis of differentially expressed genes for the TC+PKS versus TC comparison. Individual tabs show GO Biological Process and Hallmarks. Preranked GSEA performed using fgsea (minSize=10, maxSize=500) in R; DEGs from Supplementary Data 3 used a s input, ranked by log2FoldChange.

Supplementary Data 5: GSEA analysis of differentially expressed genes for the TC+S versus TC comparison. Individual tabs show GO Biological Process, Hallmarks and the clusters resulting from enrichment simplification of GO Biological Process terms shown in Figure 5b, g. Preranked GSEA p erformed as Supplementary Data 3; ontology simplification performed using simplifyEnrichment package with Wang similarity measure and mclust clustering.

Supplementary Data 6: GSEA analysis of differentially expressed genes for the TC+K versus TC comparison. Individual tabs show GO Biological Process, Hallmarks and the clusters resulting from enrichment simplification of GO Biological Process terms shown in Figure 6a, g. Data generated as described in Supplementary Data 5.

Supplementary Data 7: GSEA analysis of differentially expressed genes for the TC+P versus TC comparison. Individual tabs show GO Biological Process, Hallmarks and the clusters resulting from enrichment simplification of GO Biological Process terms shown in Figure 6i. Data generated as described in Supplementary Data 5.
