# Supplementary material for: A human model to deconvolve genotype-phenotype causations in lung squamous cell carcinoma
Source: Nat Commun. 2025 Apr 4;16:3215. doi: 10.1038/s41467-025-58343-y (PMC11971459; doi:10.1038/s41467-025-58343-y)
Supplement: Supplementary file 11 — Reporting Summary [file 41467_2025_58343_MOESM11_ESM.pdf]

nature portfolio

Corresponding author(s): Carlos Lopez-Garcia

Last updated by author(s): Jan 27, 2025

Reporting Summary

Nature Portfolio wishes to improve the reproducibility of the work that we publish. This form provides structure for consistency and transparency in reporting. For further information on Nature Portfolio policies, see our Editorial Policies and the Editorial Policy Checklist.

Statistics

For all statistical analyses, confirm that the following items are present in the figure legend, table legend, main text, or Methods section.

n/a	Confirmed	
☐	☒	The exact sample size (*n*) for each experimental group/condition, given as a discrete number and unit of measurement
☐	☒	A statement on whether measurements were taken from distinct samples or whether the same sample was measured repeatedly
☐	☒	The statistical test(s) used AND whether they are one- or two-sided<br>*Only common tests should be described solely by name; describe more complex techniques in the Methods section.*
☒	☐	A description of all covariates tested
☐	☒	A description of any assumptions or corrections, such as tests of normality and adjustment for multiple comparisons
☐	☒	A full description of the statistical parameters including central tendency (e.g. means) or other basic estimates (e.g. regression coefficient) AND variation (e.g. standard deviation) or associated estimates of uncertainty (e.g. confidence intervals)
☒	☐	For null hypothesis testing, the test statistic (e.g. *F*, *t*, *r*) with confidence intervals, effect sizes, degrees of freedom and *P* value noted<br>*Give P values as exact values whenever suitable.*
☒	☐	For Bayesian analysis, information on the choice of priors and Markov chain Monte Carlo settings
☒	☐	For hierarchical and complex designs, identification of the appropriate level for tests and full reporting of outcomes
☐	☒	Estimates of effect sizes (e.g. Cohen's *d*, Pearson's *r*), indicating how they were calculated

Our web collection on statistics for biologists contains articles on many of the points above.

Software and code

Policy information about availability of computer code

Data collection	qPCR:: Design and Analysis v1.5.2<br>Image acquisition<br>• IF Olympus VS120 Olympus: VS-ASW software V.2.9<br>• IHC: Olympus VS200: AWS version 3.4.1<br>RNAseq: NovaSeq Control Software v1.8<br>MiSeq: MiSeq FGx Control Software v1.5.0, RTA v1.18.54<br>TCGA & CPTAC: Genome Data Analysis Center (GDAC) and cBioportal (v. 6. 0. 20)<br>Western blot image acquisition: Image Lab Software v. 3.0.1.14<br>Luminiscence and fluorescence read-outs in 96-well plates: Omega 5.70 (BMG Labtech)<br>Flow Cytometry: BD FACSDiva™ Software software v. 6.1.3<br>Live-cell image acquisition: Incucyte® S3 Software (v2018B)<br>DepMap data acquisition: DepMap Public 23Q4<br>Premalignant lesion expression data: XTABLE shiny app
Data analysis	qPCR: ThermoFisher Connect (Relative Quantification App)<br>Image analysis: HALO v. 3.4 and Fiji v.2.9.0<br>RNA seq:<br>• R (version 3.6.1)<br>• Star aligner (version 2.5.1b)<br>• Adapter sequence trimming: trim_galore (version 0.6.5)

- BAM alignments were quantified in R (version 3.6.1) using the featureCounts program from the R Subread package (version 2.0.1)
- Differential expression: DESeq2 (version 1.26).
- Heatmap generation: ComplexHeatmap package (version 1.72.1)
- GSEA: fgsea (version 1.24.0)
- GO term simplification: simplifyEnrichment
- WGCNA (version 1.72-1)

Flow cytometry analysis: FloJo 10.9.0 CL
Ontology enrichment analysis: ShinyGO 0.81
GraphPad Prism 10.2.0

For manuscripts utilizing custom algorithms or software that are central to the research but not yet described in published literature, software must be made available to editors and reviewers. We strongly encourage code deposition in a community repository (e.g. GitHub). See the Nature Portfolio guidelines for submitting code & software for further information.

Data

Policy information about availability of data

All manuscripts must include a data availability statement. This statement should provide the following information, where applicable:

- Accession codes, unique identifiers, or web links for publicly available datasets
- A description of any restrictions on data availability
- For clinical datasets or third party data, please ensure that the statement adheres to our policy

The ALI culture raw RNA-seq data generated in this study are available with unrestricted access in the BioProject database under accession number: PRJNA1043668 [https://www.ncbi.nlm.nih.gov/bioproject/?term=PRJNA1043668]
The datasets that have been previously published and used in this article include:
GSE33479 [https://www.ncbi.nlm.nih.gov/geo/query/acc.cgi?acc=GSE33479] (Premalignant lesion RNA microarray data)
PDC000234 [https://proteomic.datacommons.cancer.gov/pdc/study/PDC000234] (CPTAC proteomics data)
TCGA-LUSC [https://portal.gdc.cancer.gov/projects/TCGA-LUSC] (TCGA RNA-seq data)

Research involving human participants, their data, or biological material

Policy information about studies with human participants or human data. See also policy information about sex, gender (identity/presentation), and sexual orientation and race, ethnicity and racism.

Reporting on sex and gender	Sex or gender was not considered when selecting archived formalin-fixed paraffin embedded samples human for this study as we studied differences between normal, premalignant, and invasive regions of the same samples. Therefore, the selection criteria was the presence of the three components and the results were internally controlled.<br>Sex was determined by patient (LUSC patient samples) or donor (human bronchial epithelial cells) self-report.
Reporting on race, ethnicity, or other socially relevant groupings	Race, ethnicity, or other socially relevant groupings were not considered when selecting archived formalin-fixed paraffin embedded samples human for this study as we studied differences between normal, premalignant, and invasive regions of the same samples. Therefore, the selection criteria was the presence of the three components he results were internally controlled.
Population characteristics	The samples were obtained from archived diagnostic material. Therefore, the population was not selected based on specific characteristics.
Recruitment	The samples were obtained from archived diagnostic material.
Ethics oversight	Manchester Cancer Research Centre Biobank

Note that full information on the approval of the study protocol must also be provided in the manuscript.

Field-specific reporting

Please select the one below that is the best fit for your research. If you are not sure, read the appropriate sections before making your selection.

☒ Life sciences ☐ Behavioural & social sciences ☐ Ecological, evolutionary & environmental sciences

For a reference copy of the document with all sections, see nature.com/documents/nr-reporting-summary-flat.pdf

Life sciences study design

All studies must disclose on these points even when the disclosure is negative.

Sample size	For cell biology data we used a standard n=3-4 biological replicates for three donors donor.<br>For the analysis shown in Figure 5 the number of samples was as follows: normal epithelium = 8, hyperplasia/metaplasia = 7, mild-moderate dysplasia = 4, severe dysplasia = 5 and carcinoma in situ = 5
Data exclusions	No data was excluded

Replication	For each donor we ran 3-4 biological replicates.
Randomization	Not relevant for this study as the experimental groups consisted of genotypes.<br>For the analysis in Figure 4 randomization was not possible due to the limitatation of samples with premalignant regions.
Blinding	Image analysis and bioinformatic analysis were blinded at stages that could not be carried out automatedly.

Reporting for specific materials, systems and methods

We require information from authors about some types of materials, experimental systems and methods used in many studies. Here, indicate whether each material, system or method listed is relevant to your study. If you are not sure if a list item applies to your research, read the appropriate section before selecting a response.

Materials & experimental systems

n/a	Involved in the study	
☐	☒	Antibodies
☐	☒	Eukaryotic cell lines
☒	☐	Palaeontology and archaeology
☒	☐	Animals and other organisms
☐	☒	Clinical data
☒	☐	Dual use research of concern
☒	☐	Plants

Methods

n/a	Involved in the study	
☐	☐	ChIP-seq
☐	☒	Flow cytometry
☐	☐	MRI-based neuroimaging

Antibodies

Antibodies used	p53 (SCB, sc-126) 1:1000, CDKN2A (p16) (SCB, 92803) 1:500, KEAP1 (SCB, sc-365626) 1:500, PTEN (SCB, sc-7974) 1:500, AKT (CST, 4691) 1:1000, pAKT(ser473) (CST, 4060) 1:1000, NQO1 (SCB, 32793) 1:1000, SOX2 (Abcam, ab97959) 1:1000, mCherry (CST, 43590) 1:1000, and Vinculin (Sigma, V9264) 1:10,000.<br>Secondary antibodies were goat anti-rabbit IgG HRP (Agilent Technologies, P0440801-2), or rabbit anti-mouse IgG HRP (Agilent technologies, P044701-2) at 1:5000.<br>Immunofluoresncence: anti-MUC5AC (Invitrogen, MA5-12178) (1:400) and anti-acetylated tubulin (Sigma, T6793) (1:400), anti-vimentin (Invitrogen, MA5-11883) (1:250) and anti-EPCAM (Abcam, ab223582) (1/500). Secondary antibodies included goat anti-mouse IgG1 Alexa Fluor 488 (Invitrogen, A-21121), goat anti-rabbit IgG Alexa Fluor 647 (Invitrogen, A32733), and goat anti-mouse IgG2b Alexa Fluor 647 (Invitrogen, A-21242).<br>Immunohistochemistry: anti-TTF1 (Abcam, ab76013) (1:100), anti-cytokeratin 5/6 (Invitrogen, MA191106) (1:100) and anti-p63 (Abcam, ab124762) (1:400), anti-CC10 (SCB, 365992) (1:2000), anti-SOX2 (Abcam, ab92494) (1:100), anti-mCherry (Novus, NBP2-25157) (1:500)
Validation	Western blot antibodies were validated using knowck out or knock down cell lines. Immunofluorescen and immunohistochemistry antibodies were validated based on the epithelial pattern of expression or by overexpression of the target and immunodetection in cell pellets.

Eukaryotic cell lines

Policy information about cell lines and Sex and Gender in Research

Cell line source(s)	Human bronchial epithelial cells: Lonza (details in Supplementary Table 1)<br>3T3-J2 fibroblast: Kerafast<br>Lenti X 293: Takara<br>Human Pulmonary fibroblasts: Lonza
Authentication	ALl cell lines were authenticated by STR analysis
Mycoplasma contamination	All cells were mycoplasma tested before carrying out a batch of experiments with the same frozen cell stock.
Commonly misidentified lines<br>(See ICLAC register)	N/A

Clinical data

Policy information about clinical studies

All manuscripts should comply with the ICMJE guidelines for publication of clinical research and a completed CONSORT checklist must be included with all submissions.

Clinical trial registration	*Provide the trial registration number from ClinicalTrials.gov or an equivalent agency.*

Study protocol	*Note where the full trial protocol can be accessed OR if not available, explain why.*
Data collection	*Describe the settings and locales of data collection, noting the time periods of recruitment and data collection.*
Outcomes	*Describe how you pre-defined primary and secondary outcome measures and how you assessed these measures.*

Plants

Seed stocks	N/A
Novel plant genotypes	N/A
Authentication	N/A

ChIP-seq

Data deposition

☐ Confirm that both raw and final processed data have been deposited in a public database such as GEO.

☐ Confirm that you have deposited or provided access to graph files (e.g. BED files) for the called peaks.

Data access links<br>*May remain private before publication.*	*For "Initial submission" or "Revised version" documents, provide reviewer access links. For your "Final submission" document, provide a link to the deposited data.*
Files in database submission	*Provide a list of all files available in the database submission.*
Genome browser session<br>(e.g. UCSC)	*Provide a link to an anonymized genome browser session for "Initial submission" and "Revised version" documents only, to enable peer review. Write "no longer applicable" for "Final submission" documents.*

Methodology

Replicates	*Describe the experimental replicates, specifying number, type and replicate agreement.*
Sequencing depth	*Describe the sequencing depth for each experiment, providing the total number of reads, uniquely mapped reads, length of reads and whether they were paired- or single-end.*
Antibodies	*Describe the antibodies used for the ChIP-seq experiments; as applicable, provide supplier name, catalog number, clone name, and lot number.*
Peak calling parameters	*Specify the command line program and parameters used for read mapping and peak calling, including the ChIP, control and index files used.*
Data quality	*Describe the methods used to ensure data quality in full detail, including how many peaks are at FDR 5% and above 5-fold enrichment.*
Software	*Describe the software used to collect and analyze the ChIP-seq data. For custom code that has been deposited into a community repository, provide accession details.*

Flow Cytometry

Plots

Confirm that:

☐ The axis labels state the marker and fluorochrome used (e.g. CD4-FITC).

☐ The axis scales are clearly visible. Include numbers along axes only for bottom left plot of group (a 'group' is an analysis of identical markers).

☐ All plots are contour plots with outliers or pseudocolor plots.

☐ A numerical value for number of cells or percentage (with statistics) is provided.

Methodology

Sample preparation	*Describe the sample preparation, detailing the biological source of the cells and any tissue processing steps used.*
Instrument	*Identify the instrument used for data collection, specifying make and model number.*
Software	*Describe the software used to collect and analyze the flow cytometry data. For custom code that has been deposited into a community repository, provide accession details.*
Cell population abundance	*Describe the abundance of the relevant cell populations within post-sort fractions, providing details on the purity of the samples and how it was determined.*
Gating strategy	*Describe the gating strategy used for all relevant experiments, specifying the preliminary FSC/SSC gates of the starting cell population, indicating where boundaries between "positive" and "negative" staining cell populations are defined.*

☐ Tick this box to confirm that a figure exemplifying the gating strategy is provided in the Supplementary Information.

Magnetic resonance imaging

Experimental design

Design type	*Indicate task or resting state; event-related or block design.*
Design specifications	*Specify the number of blocks, trials or experimental units per session and/or subject, and specify the length of each trial or block (if trials are blocked) and interval between trials.*
Behavioral performance measures	*State number and/or type of variables recorded (e.g. correct button press, response time) and what statistics were used to establish that the subjects were performing the task as expected (e.g. mean, range, and/or standard deviation across subjects).*

Acquisition

Imaging type(s)	*Specify: functional, structural, diffusion, perfusion.*
Field strength	*Specify in Tesla*
Sequence & imaging parameters	*Specify the pulse sequence type (gradient echo, spin echo, etc.), imaging type (EPI, spiral, etc.), field of view, matrix size, slice thickness, orientation and TE/TR/flip angle.*
Area of acquisition	*State whether a whole brain scan was used OR define the area of acquisition, describing how the region was determined.*

Diffusion MRI ☐ Used ☐ Not used

Preprocessing

Preprocessing software	*Provide detail on software version and revision number and on specific parameters (model/functions, brain extraction, segmentation, smoothing kernel size, etc.).*
Normalization	*If data were normalized/standardized, describe the approach(es): specify linear or non-linear and define image types used for transformation OR indicate that data were not normalized and explain rationale for lack of normalization.*
Normalization template	*Describe the template used for normalization/transformation, specifying subject space or group standardized space (e.g. original Talairach, MNI305, ICBM152) OR indicate that the data were not normalized.*
Noise and artifact removal	*Describe your procedure(s) for artifact and structured noise removal, specifying motion parameters, tissue signals and physiological signals (heart rate, respiration).*
Volume censoring	*Define your software and/or method and criteria for volume censoring, and state the extent of such censoring.*

Statistical modeling & inference

Model type and settings	*Specify type (mass univariate, multivariate, RSA, predictive, etc.) and describe essential details of the model at the first and second levels (e.g. fixed, random or mixed effects; drift or auto-correlation).*
Effect(s) tested	*Define precise effect in terms of the task or stimulus conditions instead of psychological concepts and indicate whether ANOVA or factorial designs were used.*

Specify type of analysis: ☐ Whole brain ☐ ROI-based ☐ Both

Statistic type for inference

(See Eklund et al. 2016)

Specify voxel-wise or cluster-wise and report all relevant parameters for cluster-wise methods.

Correction

Describe the type of correction and how it is obtained for multiple comparisons (e.g. FWE, FDR, permutation or Monte Carlo).

Models & analysis

n/a	Involved in the study
☐	☐ Functional and/or effective connectivity
☐	☐ Graph analysis
☐	☐ Multivariate modeling or predictive analysis

Functional and/or effective connectivity

Report the measures of dependence used and the model details (e.g. Pearson correlation, partial correlation, mutual information).

Graph analysis

Report the dependent variable and connectivity measure, specifying weighted graph or binarized graph, subject- or group-level, and the global and/or node summaries used (e.g. clustering coefficient, efficiency, etc.).

Multivariate modeling and predictive analysis

Specify independent variables, features extraction and dimension reduction, model, training and evaluation metrics.
